# Comparison of Pattern Discrimination Mechanisms of Hebbian and Spatiotemporal Learning Rules in Self-Organization

**DOI:** 10.3389/fnsys.2021.624353

**Published:** 2021-03-29

**Authors:** Hiromichi Tsukada, Minoru Tsukada

**Affiliations:** ^1^College of Engineering, Chubu University, Kasugai, Japan; ^2^Neural Computation Unit, Okinawa Institute of Science and Technology Graduate University, Okinawa, Japan; ^3^Brain Science Institute, Tamagawa University, Tokyo, Japan

**Keywords:** spetiotemporal learning rule, STLR, contextual learning, context-dependent memory, self-organization, pattern discrimination, pattern completion, Hebbian learning rule

## Abstract

The spatiotemporal learning rule (STLR) proposed based on hippocampal neurophysiological experiments is essentially different from the Hebbian learning rule (HEBLR) in terms of the self-organization mechanism. The difference is the self-organization of information from the external world by firing (HEBLR) or not firing (STLR) output neurons. Here, we describe the differences of the self-organization mechanism between the two learning rules by simulating neural network models trained on relatively similar spatiotemporal context information. Comparing the weight distributions after training, the HEBLR shows a unimodal distribution near the training vector, whereas the STLR shows a multimodal distribution. We analyzed the shape of the weight distribution in response to temporal changes in contextual information and found that the HEBLR does not change the shape of the weight distribution for time-varying spatiotemporal contextual information, whereas the STLR is sensitive to slight differences in spatiotemporal contexts and produces a multimodal distribution. These results suggest a critical difference in the dynamic change of synaptic weight distributions between the HEBLR and STLR in contextual learning. They also capture the characteristics of the pattern completion in the HEBLR and the pattern discrimination in the STLR, which adequately explain the self-organization mechanism of contextual information learning.

## 1. Introduction

Learning is the embedding of information from the outside world into the changes in the connections between neurons in a neural network (changes in synaptic weights) based on the correlation of neural activity. The representation of information in neural networks through learning is called self-organization. In general, learning a neural network requires setting all synaptic weights in the network to initial values before training. The synaptic weight is generally initialized with a random number. Therefore, when the synapse weight vector of each neuron is normalized, it is converted into a unit vector in an n-dimensional space and uniformly distributed near the hypersphere. In contrast, the input vectors in the neural network are usually not evenly distributed and are grouped into relatively small sections of the hypersphere surface. Considering the case where the input series are dynamically input from the external environment, each input vector in the series will be similar to each other. These input vectors are discriminated into different categories or integrated into one category in the information processing of memory in the brain. Therefore, these two contradictory functions are working in the memory system of the brain. Here we discuss the possibility that the spatiotemporal learning rule (STLR) and Hebbian learning rule (HEBLR) have such a functional role in memory and learning processing, and we clarify their characteristics.

The HEBLR (Hebb, 1949) modifies neural network connections according to the instantaneous external information when an output neuron fires. That is, if the output neuron does not fire, nothing occurs. This learning rule is widely used in unsupervised learning in neural networks (Widrow, 1959; Rosenblatt, 1962; Grossberg, 1974; Kohonen, 1988). In contrast, the STLR proposed by Tsukada et al. enables learning according to the information structure of the external environment without firing the output neurons (Tsukada et al., 1996, 2007; Tsukada and Pan, 2005). This enables a significantly flexible representation of information. The difference between the two learning rules was clarified using a simulation model of a neural network trained with relatively similar spatiotemporal pattern sequences (Tsukada and Pan, 2005). The HEBLR has the property of completing multiple similar input sequences into a single output pattern, whereas the STLR can discriminate multiple similar input sequences into separate output patterns. That is, the HEBLR has a pattern completion function and STLR has a pattern discrimination function for similar context input pattern sequences.

This study explains the difference between the two rules based on the self-organization mechanisms of the training vector and the synaptic weight vector. The clarification of the mechanism helps us to understand the use of the learning rule based on physiological experiments for the representation of information in learning and memory networks and also contributes to the applications in brain-inspired artificial intelligence.

## 2. Methods

### 2.1. Neuron Model

**Figure 1:**
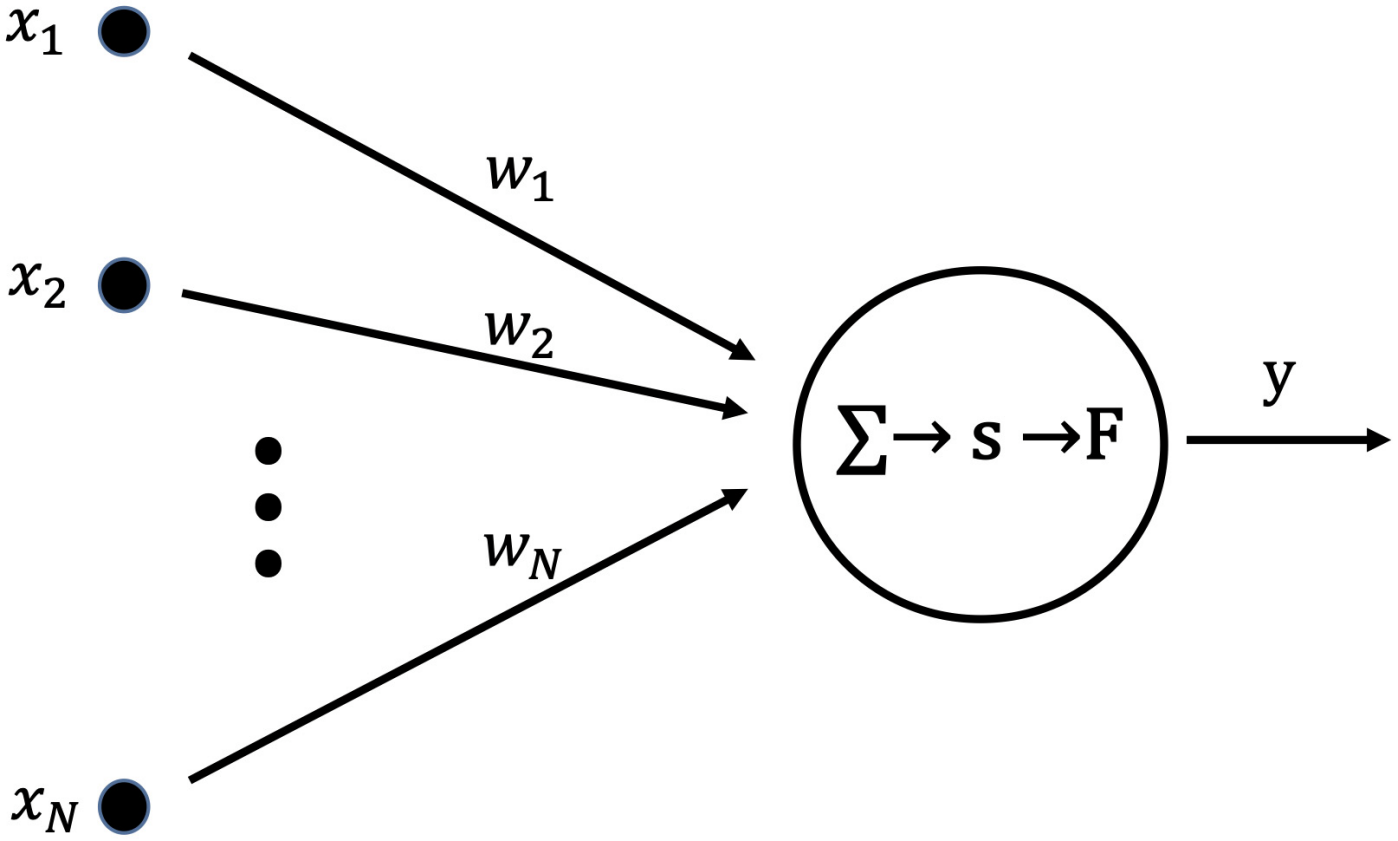
Artificial neuron used as the fundamental building block for single layer neural networks. The internal state, *s*, is calculated as the product of the input vector, *X*, and the weight vector, *W*. Output *y* is obtained by applying the output function, *F*, to the internal state, *s*.

Figure 1 shows a neuron used as the basic unit of a learning neural network. The set of inputs, *X* = (*x*_1_, …, *x*_*N*_), from an external network or existing networks is applied to a post-synaptic neuron. Each *x* is multiplied by the synaptic weight, *w*, and summed. Then, the postsynaptic membrane potential, *s*, is given by the following equation:

(1) $$\begin{array}{l} s=w_{1}x_{1}+w_{2}x_{2}+\cdots +w_{N}x_{N}. \end{array}$$

Here, the output *y* is given by the following equation with the output function, F, and the threshold value, η:

(2) $$\begin{array}{l} y=F\left(s-\eta \right), \end{array}$$

(3) $$\begin{array}{l} F\left(x\right)=\{\begin{array}{cc} 1 & x\geq 0\\ 0 & x<0. \end{array} \end{array}$$

### 2.2. Network Architecture

**Figure 2:**
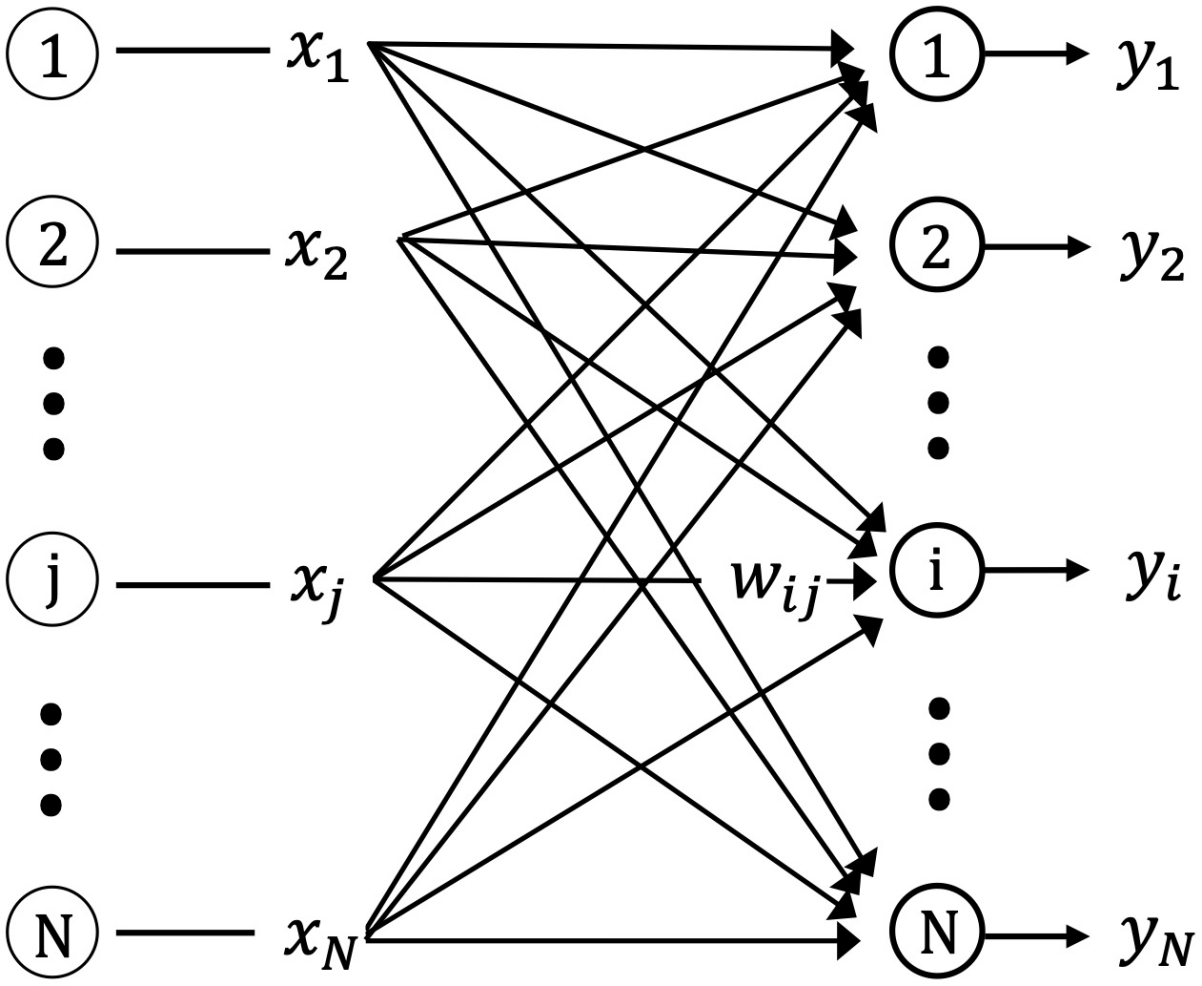
Schematic diagram of the single layer feed-forward neural network. *x*_*j*_ is the input from pre-synaptic neurons, *y*_*i*_ is the output of post-synaptic neurons, and *w*_*ij*_ is the connection weight matrix between pre-synaptic and post-synaptic neurons.

Figure 2 shows the one-layer neural network used in this study for storing the spatiotemporal pattern (sequences of input vectors) using unsupervised learning. The network is composed of *N* neurons with feed-forward excitatory connections. Each neuron is connected to the source (input) neurons via excitatory synapses. The input neuron, *j*(*j* = 1, 2, …, *N*), connects the output neuron, *i*(*i* = 1, 2, …, *N*), via the synaptic weight, *w*_*ij*_.

The input signals at time *t*_*k*_, *X*(*t*_*k*_), consist of a spatial pattern with the n dimensional binary elements, (*x*_1_, *x*_2_, …, *x*_*N*_). For example, a neuron, *i*, with a synapse weight vector, *W*_*i*_(*t*_*k*_), for an input vector, *X*(*t*_*k*_), at a certain time, *t*_*k*_, is shown in Figure 2. Neuron *i* has the sequence of synapse weight vectors {*W*_*i*_(*t*_1_), *W*_*i*_(*t*_2_), …, *W*_*i*_(*t*_*k*_)} for the input vector sequence, {*X*(*t*_1_), *X*(*t*_2_), …, *X*(*t*_*k*_)}. *s*_*i*_(*t*_*k*_) is expressed by the following equation for the internal state of neuron *i* at time *t*_*k*_:

(4) $$\begin{array}{l} s_{i}\left(t_{k}\right)=\overset{N}{\underset{j=1}{\sum }}w_{ij}\left(t_{k}\right)x_{j}\left(t_{k}\right)=W_{i}\left(t_{k}\right)X\left(t_{k}\right). \end{array}$$

This value is calculated for each neuron, *i*, in the network. Following the calculation for *s*_*i*_(*t*_*k*_), the output function, *F*, and the threshold, η, is used to yield the output, *y*(*t*_*k*_), using the following equations:

(5) $$\begin{array}{l} y_{i}\left(t_{k}\right)=F\left(s_{i}\left(t_{k}\right)-\eta \right), \end{array}$$

(6) $$\begin{array}{l} F\left(x\right)=\{\begin{array}{cc} 1 & x\geq 0\\ 0 & x<0. \end{array} \end{array}$$

### 2.3. Input Spatiotemporal Pattern

**Figure 3:**
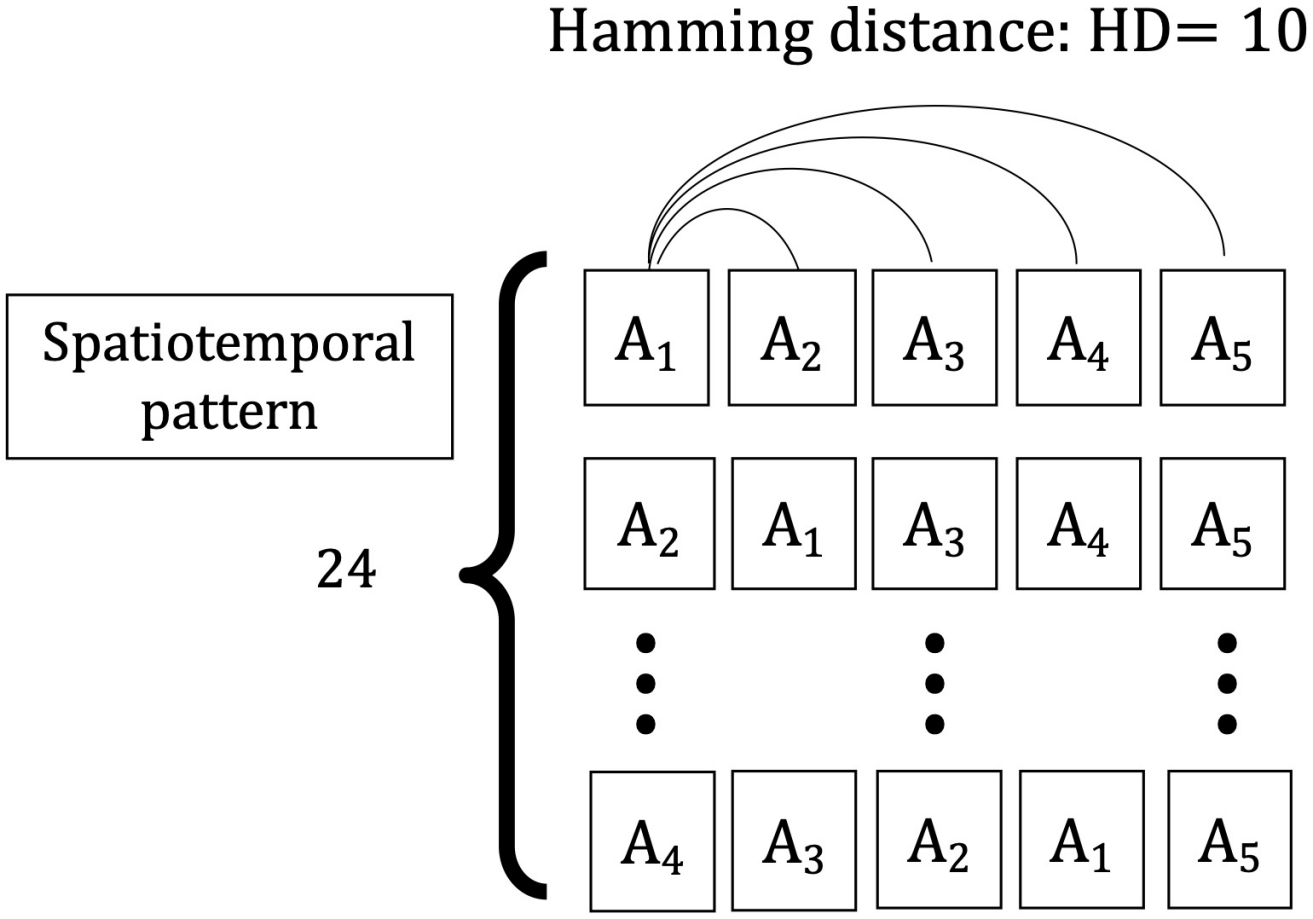
Spatiotemporal patterns applied to the single-layer network. A sample of the spatial frame, i.e., *A*_1_, consisting of *N* = 120 elements. Spatiotemporal patterns of contextual inputs, {*X*(*t*_1_), *X*(*t*_2_), …, *X*(*t*_5_)}, each pattern has a temporal sequence of 5 spatial patterns {*A*_1_, *A*_2_, *A*_3_, *A*_4_, *A*_5_}. The Hamming distance between every two spatial patterns is 10 bits.

The spatiotemporal pattern used in this simulation is a sequence of five spatial patterns {*A*_1_, *A*_2_, *A*_3_, *A*_4_, *A*_5_} with a Hamming distance (*HD* = 10) between them, comprising input vector sequences {*X*(*t*_1_), *X*(*t*_2_), …, *X*(*t*_5_)}. The vectors consist of n elements (*N* = 120), with each element randomly selected as “1” or “0” and the total number of “1” s is constant over different spatial patterns. This value is constant (for this simulation, half of the elements of one vector are “1” and half are “0”). To evaluate the learning of the input information learned in the weight space owing to the change of the series, two types of input series vectors are considered as training sets: one is each sequence having the same spatial patterns, {*X*(*t*_1_) = *X*(*t*_2_) = *X*(*t*_3_) = *X*(*t*_4_) = *X*(*t*_5_) = *A*_*i*_|*i* ∈ (1, …, 5)}, and the other is the sequences having the different spatial patterns, which consist of four randomly chosen elements from five spatial patterns {*A*_1_, *A*_2_, *A*_3_, *A*_4_, *A*_5_} and replaced the first four input vectors {*X*(*t*_1_), *X*(*t*_2_), *X*(*t*_3_), *X*(*t*_4_)} in each sequence (Figure 3). The two types of sequences are applied to the network as a training set, and the weight vector distributions for the HEBLR and STLR are compared.

### 2.4. Learning Rules: HEBLR and STLR

#### 2.4.1. HEBLR Algorithm

Hebb (1949) proposed that a synaptic connection between the input and output neurons is modified whenever both neurons fire. This is considered to strengthen a synapse according to the correlation between the neuron excitation levels of both neurons. This is expressed by the following equation:

(7) $$\begin{array}{l} w_{ij}\left(t_{k+1}\right)=w_{ij}\left(t_{k}\right)+\alpha y_{i}\left(t_{k}\right)x_{j}\left(t_{k}\right). \end{array}$$

The synaptic weight change of HEBLR is given by

(8) $$\begin{array}{l} w_{ij}\left(t_{k+1}\right)=\{\begin{array}{cc} w_{ij}\left(t_{k}\right)+\delta w & s_{i}\left(t_{k}\right)\geq \eta \\ w_{ij}\left(t_{k}\right) & s_{i}\left(t_{k}\right)<\eta . \end{array} \end{array}$$

Here, *w*_*ij*_(*t*_*k*_) is the synaptic weight from neuron *j* to neuron *i* at time *t*_*k*_, and *w*_*ij*_(*t*_*k*+1_) is the weight at time *t*_*k*+1_. *x*_*j*_(*t*_*k*_) is the activity of the *j*th input neuron at time *t*_*k*_, *y*_*i*_(*t*_*k*_) is the activity of the *i*th output neuron at time *t*_*k*_, and α is the training rate coefficient. *s*_*i*_(*t*_*k*_) is the internal state of neuron *i* at time *t*_*k*_. The weight change, *w*_*ij*_(*t*_*k*+1_) − *w*_*ij*_(*t*_*k*_) = δ*w*(*constant*), occurs under the condition of *y*_*i*_(*t*_*k*_) = 1 and *x*_*j*_(*t*_*k*_) = 1. *y*_*i*_(*t*_*k*_) = 1 is given by Equation (5) in the case *s*_*i*_(*t*_*k*_) ≥ η.

#### 2.4.2. STLR Algorithm

Changes in the synaptic weights in the STLR depend on “timing coherence” and “temporal history” of the input neuronal activity without firing of output neurons (Tsukada et al., 1994, 1996, 2007). The time history factor was omitted here for simplicity. The coincidence coefficient (Fujii et al., 1996), *I*_*ij*_(*t*_*k*_), depends on the strength of the cooperative activity of the other input neurons when the spike of input neuron *j* reaches output neuron *i* at time *t*_*k*_ and is defined as follows:

(9) $$\begin{array}{l} I_{ij}\left(t_{k}\right)=w_{ij}\left(t_{k}\right)x_{j}\left(t_{k}\right)\overset{N}{\underset{m=1,m\neq j}{\sum }}w_{im}\left(t_{k}\right)x_{m}\left(t_{k}\right). \end{array}$$

Here, under conditions where the network size is large and the firing of the incoming inputs is not sparse, the summation term is given by

(10) $$\begin{array}{l} \begin{array}{c} \overset{N}{\underset{m=1,m\neq j}{\sum }}w_{im}\left(t_{k}\right)x_{m}\left(t_{k}\right)=s_{i}\left(t_{k}\right)-w_{ij}\left(t_{k}\right)x_{j}\left(t_{k}\right)≃s_{i}\left(t_{k}\right),\\ ∵s_{i}\left(t_{k}\right)\gg w_{ij}\left(t_{k}\right)x_{j}\left(t_{k}\right). \end{array} \end{array}$$

Therefore, *I*_*ij*_(*t*_*k*_) is approximated as follows:

(11) $$\begin{array}{l} I_{ij}\left(t_{k}\right)=w_{ij}\left(t_{k}\right)x_{j}\left(t_{k}\right)s_{i}\left(t_{k}\right). \end{array}$$

The algorithm for changing synaptic weights by the STLR is given by

(12) $$\begin{array}{l} w_{ij}\left(t_{k+1}\right)=\{\begin{array}{cc} w_{ij}\left(t_{k}\right)+\delta w & I_{ij}\left(t_{k}\right)\geq \theta_{1}\\ w_{ij}\left(t_{k}\right) & \theta_{1}>I_{ij}\left(t_{k}\right)>\theta_{2}\\ w_{ij}\left(t_{k}\right)-\delta w & I_{ij}\left(t_{k}\right)\leq \theta_{2}. \end{array} \end{array}$$

Here, θ_1_ and θ_2_ are determined by the change in the intracellular *Ca*^2+^ concentration due to its influx through the N-Methyl-D-aspartate (NMDA) channel and release from the intracellular stores with high density inducing long-term potentiation (LTP) and low density inducing long-term depression (LTD); this is called the Bienenstock-Cooper-Munro (BCM) synapse modification rule (Bienenstock et al., 1982; Lacaille and Schwartzkroin, 1988).

## 3. Comparison of Learning Algorithms: HEBLR and STLR

### 3.1. HEBLR Algorithm

The synaptic weight change ΔŴ_*i*_(*t*_*k*_) of neuron *i* depends on the number of the increasing weight *N*_*i*_(*t*_*k*_) when *y*_*i*_(*t*_*k*_) = 1 in the HEBLR. The equation is given by

(13) $$\begin{array}{l} \Delta {Ŵ}_{i}\left(t_{k}\right)=\left\{y_{i}\left(t_{k}\right)\underset{j}{\sum }x_{j}\left(t_{k}\right)\right\}\delta w=N_{i}\left(t_{k}\right)\delta w. \end{array}$$

Neurons with weight vector *W*_*i*_(*t*_*k*_) that are significantly correlated with the input vector *X*(*t*_*k*_) are trained to reduce the difference between their weight vector and the input vector.

If the element vectors of the input vector series are identical {*X*(*t*_1_) = *X*(*t*_2_) = *X*(*t*_3_) = *X*(*t*_4_) = *X*(*t*_5_) = *A*_*i*_|*i* ∈ (1, …, 5)}, the group of neurons that satisfy *s*_*i*_(*t*_*k*_) ≥ η is the “winner” and the weights associated with the winning neuron are enhanced with training to yield the same output vector. Each distribution of the synapse weight vector has a one-peak distribution to approach the input vector, and the distribution sharpens as the training progresses. In contrast, for an input vector sequence whose differences between the input vectors have *HD* = 10, the common elements among the input vectors are strengthened, whereas the different elements among the input vectors are subdued as training progresses. Thereafter, the distribution of the synaptic weight vector converges to a one-peak distribution based on the common elements between the input vectors.

### 3.2. STLR Algorithm

The important point of STLR is that the amount of change in *w*_*ij*_ differs depending on the classification of *I*_*ij*_ by two different thresholds, θ_1_ and θ_2_, which enables various learning. In relation to the input, *x*_*j*_, STLR learning proceeds only when *x*_*j*_ = 1 (see Equation (11)). Then, the equation is given by

(14) $$\begin{array}{l} {Î}_{ij}\left(t_{k}\right)=w_{ij}\left(t_{k}\right)s_{i}\left(t_{k}\right). \end{array}$$

This simplification helps us to understand how *w*_*ij*_ changes with learning, depending on the threshold. The two quantities mentioned above (*I*_*ij*_ and *w*_*ij*_) are quite difficult for us to understand intuitively because the changes in their quantities due to learning are diverse (in position, orientation, and magnitude) in high-dimensional space. Therefore, we try to capture the whole picture of these quantities by considering a transformation with a one-to-one correspondence that projects these quantities into the interior of the unit sphere in high-dimensional space. This would allow us to visualize the variation of *w*_*ij*_ and capture the important aspects of the change due to learning. According to this line of thinking, we normalize the input vector by its norm as follows:

(15) $$\begin{array}{l} {\tilde{x}}_{j}=\frac{x_{j}}{\left||X|\right|}, \end{array}$$

where

(16) $$\begin{array}{l} X=\left(x_{1},x_{2},\ldots ,x_{j},\ldots ,x_{N}\right). \end{array}$$

Similarly, normalization of the synaptic weight vector is

(17) $$\begin{array}{l} {\tilde{w}}_{ij}=\frac{w_{ij}}{\left||W_{i}|\right|}, \end{array}$$

where

(18) $$\begin{array}{l} W_{i}=\left(w_{i1},w_{i2},\ldots ,w_{ij},\ldots ,w_{iN}\right). \end{array}$$

Then, normalization of the internal state of neuron *i* is given by

(19) $$\begin{array}{l} {\tilde{s}}_{i}=\overset{N}{\underset{j=1}{\sum }}{\tilde{w}}_{ij}{\tilde{x}}_{j}. \end{array}$$

By the above normalization, Î_*ij*_(*t*_*k*_) in Equation (14) is transformed into the interior of the unit sphere and on its surface, and is given by the following equation with two different thresholds, ${\tilde{\theta }}_{1}$ and ${\tilde{\theta }}_{2}$:

(20) $$\begin{array}{l} {Ĩ}_{ij}\left(t_{k}\right)={\tilde{w}}_{ij}\left(t_{k}\right){\tilde{s}}_{i}\left(t_{k}\right). \end{array}$$

**Figure 4:**
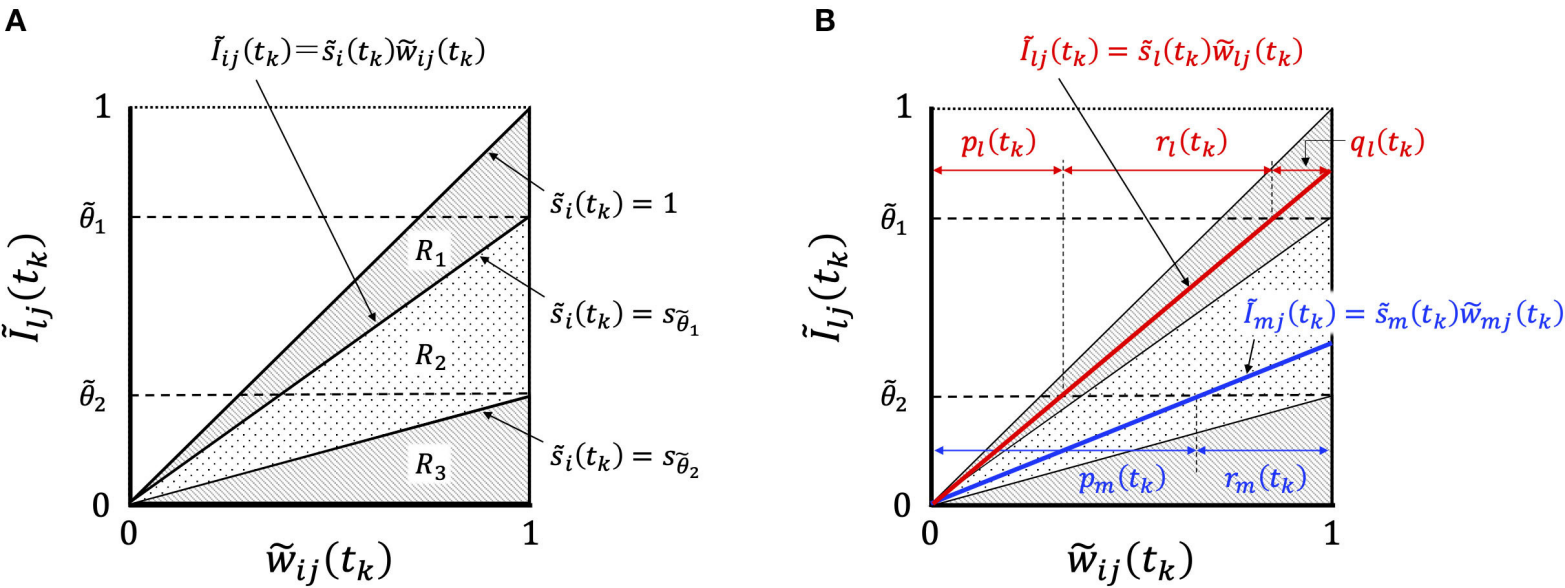
Graphical representation of synaptic weight changes by the STLR algorithm. **(A)** Region *R* is partitioned into three regions depending on the magnitude of ${\tilde{s}}_{i}\left(t_{k}\right)$; *R*_1_ consists of three regions of “enhancement,” “invariance,” and “attenuation,” *R*_2_ of two regions of “invariance” and “attenuation” and *R*_3_ of one region of “attenuation.” **(B)** The relationship between the synaptic weights ${\tilde{w}}_{ij}\left(t_{k}\right)$ and Ĩ_*ij*_(*t*_*k*_) in Equation (20). In the case of the relation belonging to the region *R*_1_ shown by red line, the weight change has “enhancement,” *q*_*l*_(*t*_*k*_), “invariance,” *r*_*l*_(*t*_*k*_), and “attenuation,” *p*_*l*_(*t*_*k*_). The weight vector ${\tilde{W}}_{l}\left(t_{k}\right)$ is trained in the direction of reducing the difference between the weight vector and the input vector according to the magnitude of *q*_*l*_(*t*_*k*_). In the case of the relation belonging to the region *R*_2_, as shown by blue line, the weight change has “invariance,” *r*_*m*_(*t*_*k*_), and “attenuation,” *p*_*m*_(*t*_*k*_). The weight vector ${\tilde{W}}_{m}\left(t_{k}\right)$ is not trained and remains unused.

In order to consider the essential features of STLR, we focused on the relationship between Ĩ_*ij*_(*t*_*k*_) and ${\tilde{w}}_{ij}\left(t_{k}\right)$, and showed the change in their relationship with learning in Figure 4.

The relationship between the synaptic weights ${\tilde{w}}_{ij}\left(t_{k}\right)$ and Ĩ_*ij*_(*t*_*k*_) in Equation (20) is represented by straight lines passing through the origin of the coordinate axes with the gradient ${\tilde{s}}_{i}\left(t_{k}\right)$. The synaptic weights ${\tilde{w}}_{ij}\left(t_{k+1}\right)$ can be classified into three types (*R*_1_, *R*_2_, and *R*_3_) of weight changes by the magnitude of ${\tilde{s}}_{i}\left(t_{k}\right)$: *R*_1_, which satisfies the condition ${\tilde{s}}_{i}\left(t_{k}\right)\geq {\tilde{\theta }}_{1}$, has the three components “enhancement,” “invariance,” and “attenuation”; *R*_2_ $\left({\tilde{\theta }}_{1}>{\tilde{s}}_{i}\left(t_{k}\right)>{\tilde{\theta }}_{2}\right)$ has the two components “invariance” and “attenuation”; and *R*_3_ $\left({\tilde{s}}_{i}\left(t_{k}\right)\leq {\tilde{\theta }}_{2}\right)$ has a component “attenuation” (Figure 4A). We defined the group of weight changes, “enhancement,” *q*_*i*_, “invariance,” *r*_*i*_, and “attenuation,” *p*_*i*_, which are normalized by the norm of weights for neuron *i*. When the normalized values at time *t*_*k*_ are *q*_*i*_(*t*_*k*_), *r*_*i*_(*t*_*k*_), and *p*_*i*_(*t*_*k*_), respectively, then *q*_*i*_(*t*_*k*_) + *r*_*i*_(*t*_*k*_) + *p*_*i*_(*t*_*k*_) = 1. We explain the mechanisms of synaptic weight changes in Figure 4B.

When a series of input vectors are in the same context, {*X*(*t*_1_) = *X*(*t*_2_) = *X*(*t*_3_) = *X*(*t*_4_) = *X*(*t*_5_) = *A*_*i*_|*i* ∈ (1, …, 5)}, then the distribution of the synaptic weights changes dynamically as the training progresses. The synaptic weights of neurons are enhanced by the same inputs each time, yielding the same output vector. The synapse weight vectors are rotated in the direction of the input vector and produce a one-peak distribution around the input vector. In this case, the characteristics are similar to those of the HEBLR.

In the case of the different input vector series (different context) whose differences among the input vectors have *HD* = 10, the synapse weight vectors are trained by the different inputs; the distribution is dependent on each input vector because each synaptic weight vector is trained by different input vectors. Therefore, the distribution becomes multimodal depending on the different input vectors, which is completely different from the distribution in the HEBLR.

To conclude, we assume that the distribution of synaptic weight vectors related to the output has similar characteristics to the synaptic weight vector distribution of HEBLR for learning the same context. However, the synaptic weight vectors are trained near the axis of different input vectors in learning different contexts. Thereafter, the distribution of their weight vectors is developed near each input vector. Therefore, the distribution of synaptic weight vectors is multi-modal, which is completely different from the distribution of synaptic weight vectors in HEBLR.

## 4. Simulation Results

To investigate the difference between the HEBLR and STLR for a context input, we put the input series generated by combining spatial patterns (see section 2.3) into the network. We set the initial synaptic weights to be a uniform distribution, and compared the distributions after learning using each learning rule. To examine the effect of context learning, we considered two types of inputs: the same contextual input series {*X*(*t*_1_) = *X*(*t*_2_) = *X*(*t*_3_) = *X*(*t*_4_) = *X*(*t*_5_) = *A*_1_} and different contextual input series {*X*(*t*_1_) = *A*_1_, *X*(*t*_2_) = *A*_2_, *X*(*t*_3_) = *A*_3_, *X*(*t*_4_) = *A*_4_, *X*(*t*_5_) = *A*_5_}.

### 4.1. Learning With Same Contextual Input Sequences

**Figure 5:**
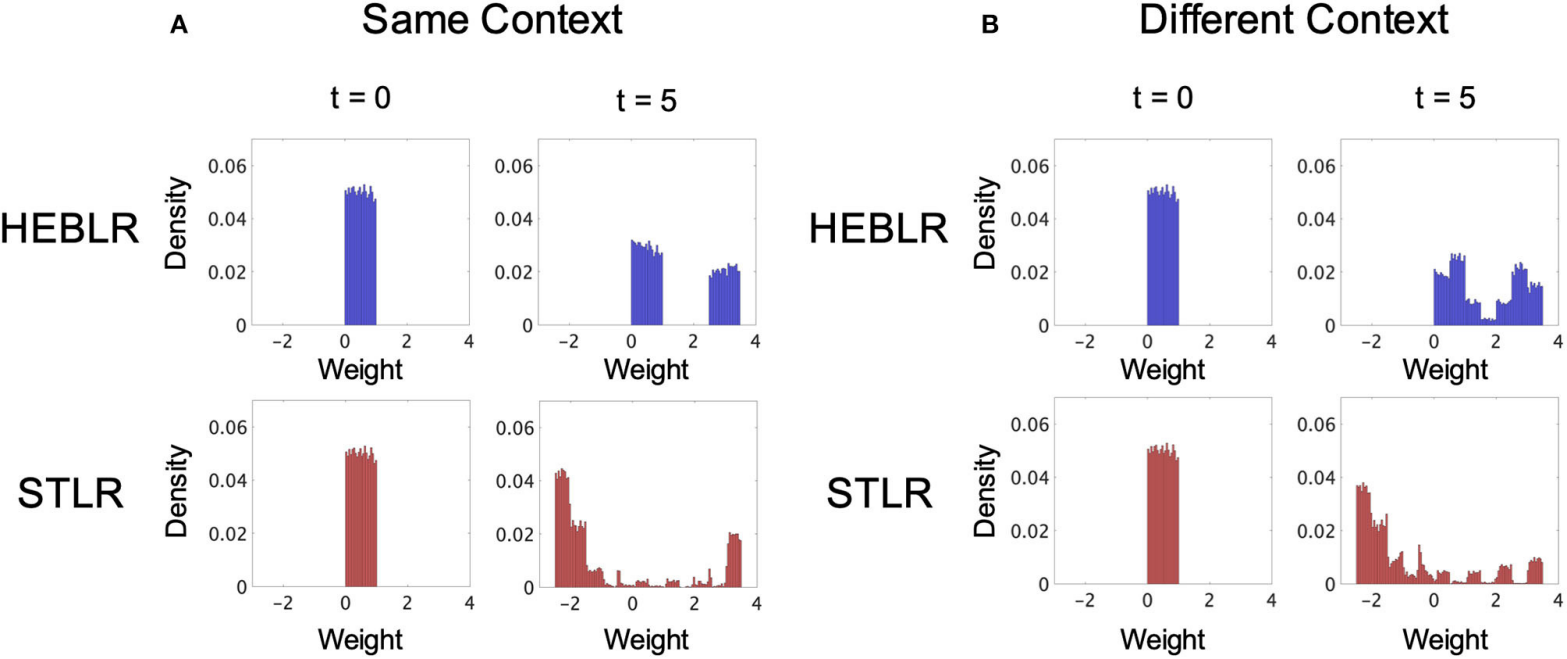
Simulation results of the synaptic weight vector distributions of HEBLR and STLR before training (*t* = 0), and after 5 steps (*t* = 5). **(A)** Weight distribution for HEBLR and STLR in the same contextual input. Initial distribution of synaptic weights for HEBLR and STLR (left upper and lower panels). Weight distribution after 5 steps in HEBLR (right upper panel). Weight distribution after 5 steps in STLR (right lower panel). **(B)** Weight distribution for HEBLR and STLR in different contextual inputs. Initial distribution of synaptic weights for HEBLR and STLR (left upper and lower panels). Weight distribution after 5 steps in HEBLR (right upper panel). Weight distribution after 5 steps in STLR (right lower panel).

Figure 5A shows the synaptic weight distributions using the HEBLR (upper panel) and STLR (lower panel) for the same contextual input sequences.

The distribution of synaptic weights in the HEBLR represents a uniform distribution in the initial step (*t* = 0; upper left panel in Figure 5A) and a bimodal distribution after five steps (*t* = 5; upper right panel in Figure 5A). The simulation result is consistent with the results of the HEBLR algorithm described in section 3.1. The “enhancement” subset of synaptic weight vectors changes in the direction of the input vector after training and exhibits a unimodal distribution. The “invariant” subset remains untrained and appears as another unimodal distribution. The synaptic weights that were initially associated with output firing continue to be enhanced, whereas the other synapse weights remain unchanged. Therefore, the output vector of the network after five steps is similar to the vector enhanced by the initial firing.

In the STLR training, the distribution of synaptic weights represents a uniform distribution in the initial step (*t* = 0; lower left panel in Figure 5A) and a bimodal distribution after five steps (*t* = 5; lower right panel in Figure 5A). The bimodal distributions are mainly created by the “enhancement” subset trained in the direction of the input vectors and the “attenuation” subset trained the opposite direction of the input vectors. These two distributions become larger in the case of same contextual input sequences because the same input vector trains the same synaptic weight many times. Our simulation results also show a small distribution created by the “invariant” subset in between the two distributions (Figure 5A, lower right panel).

### 4.2. Learning With Different Contextual Input Sequences

Figure 5B shows the synaptic weight distributions using the HEBLR (upper panel) and STLR (lower panel) for the different contextual input sequences.

The distribution of synaptic weights in the HEBLR represents a uniform distribution in the initial step (*t* = 0; upper left panel in Figure 5B) and a bimodal distribution after five steps (*t* = 5; upper right panel in Figure 5B). The bimodal distribution represented the synaptic weights trained by the “enhancement” and “invariant” subsets, as discussed in section 3.1. Therefore, the weight distribution is similar to the weight distribution trained by the same context input (upper right panel in Figure 5A).

The distribution of synaptic weights in the STLR represents the multimodal distribution after five steps (*t* = 5; lower right panel in Figure 5B). This distribution differs from the distribution in the same contextual input sequences. There are mainly two large peaks in the positive and negative regions in the same contextual input sequences (lower right panel in Figure 5A), whereas there are the multiple peaks in the case of the different contextual input sequences (lower right panel in Figure 5B). These multiple peaks can be interpreted as the effect of learning by the different five inputs vectors. When different contextual input sequences are trained, the “enhancement” subset creates new distributions around each of the five different input vectors. Therefore, new synaptic weights corresponding to each input are enhanced and form multiple peaks. The two large peaks (positive and negative regions) corresponding to the “enhancement” and “attenuation” subsets created by the same input sequences decrease in size in the different contextual input sequences.

### 4.3. Comparison Between the HEBLR and STLR in the Same and Different Contextual Input Sequences

We compared the synaptic weight distributions trained by the HEBLR in the same and different contextual input sequences (upper right panel in Figures 5A,B). The synaptic weights associated with output firing initially continue to be enhanced (“enhancement” subset), whereas the other synapse weights remain unchanged (“invariant” subset) for both identical and different context input sequences. This characteristic in the HEBLR produces the bimodal distribution after five steps in both conditions. These results are consistent with the HEBLR algorithm described in section 3.1.

We also compared the synaptic weight distributions trained by the STLR in the same and different contextual input sequences (Figures 5A,B). The distribution of synaptic weights represents a bimodal distribution after five steps in the same contextual input sequences (lower right panel in Figure 5A) and a multimodal distribution after five steps in the different contextual input sequences (lower right panel in Figure 5B). The bimodal distributions in the same contextual input sequences are mainly created by the “enhancement” subset trained in the direction of the input vectors and the “attenuation” subset trained the opposite direction of the input vectors. These two distributions become larger because the same input vector trained the same synaptic weight several times. The distribution created by the “invariant” subset is also in between the two distributions; however, the size is quite small. In contrast, the multimodal distributions in the different contextual input sequences are also trained by the “enhancement” and “attenuation” subsets. However, the difference from the distribution in the same contextual input sequences is in that the “enhancement” subset creates new distributions around each of the five different input vectors. This mechanism produces the multiple peaks. Therefore, the two large peaks (positive and negative regions) corresponding to the “enhancement” and “attenuation” subsets created by the same input sequences decreased in size, and the synaptic weights form the multimodal distribution in the different contextual input sequences.

Theoretically, the synaptic weights are trained by three types of conditions (“enhancement,” “invariant,” and “attenuation”) in the STLR, and our simulation results were consistent with those of the algorithm described in section 3.2.

## 5. Discussions

Life improves itself based on the structure of the outside world, or rather, it would be better to say “creates”. This is self-organization and the basic principle of life. How the brain achieves this is a basic secret of the brain. Here, we focused on two learning rules (HEBLR and STLR), that have been confirmed in physiological experiments, in excitatory feed-forward neural networks, and clarified the differences of these two learning rules in the self-organization mechanisms for learning dynamic spatiotemporal information from the external world.

Hebb's rule modifies the synaptic connections according to the instantaneous external information when output neurons are fired. Therefore, if they do not fire, the synaptic connections do not change anything. In contrast, the STLR enables modifying the synaptic connections according to the external environment without firing the output neurons and transforming the spatio-temporal input information into the synaptic weight space. That is, the STLR enables adaption to the information structure of the external world without requiring the self-ignition of neurons. This function develops a significantly flexible memory structure. Both learning rules are working in the hippocampus brain area (Tsukada et al., 1996, 2005, 2007).

We assumed the case that events in the natural world are related to each other in time. For example, we have to process information from the external environment dynamically when driving a car. If we made an error in the order of pressing the brake and the accelerator, this error can cause a serious accident. Therefore, it is important to consider how we can learn similar spatiotemporal contextual information that changes dynamically over time. To answer this question, we investigated the learning algorithms of HEBLR and STLR to identify their self-organization mechanism using relatively similar spatiotemporal context information, and we clarified the differences between them through our simulations. Here, we discuss the self-organization mechanisms of the two learning rules (HEBLR and STLR) from both the algorithm and simulation aspects.

### 5.1. Learning Algorithms

In the HEBLR, a group of cells fires when the inner product of the input vector and the weight vector exceeds the threshold, η; then, the weights associated with the fired cells are strengthened. This means that the group of cells with weights similar to those of the input vector is selected and further strengthened with learning each time. The weight vector changes in direction to decrease the distance from the input vector. That is, the weight vector is rotated in the direction of the input vector without significant changes in its size. In the HEBLR, cells with a weight vector that has a low correlation with the input vector do not fire. Consequently, these cells do not produce any output and do not learn.

The STLR learns input information, synchronization, and the degree of coincidence between input vectors. The coincidence coefficient depends on the inner product of the input vector and the synapse weight vector (Equation 9). Then, the threshold value, θ_1_ and θ_2_, determines the increase or decrease of the synaptic weights (Equation 11). The synapse weight vector is uniformly and randomly distributed before learning and develops its weight distribution depending on the input vector sequence. The synaptic weight vectors in the STLR are created in mainly three distributions: a distribution with a peak around the input vector, one with a peak around the inversion vector of the input vector, and one with a peak in between them.

If the element vectors of the input vector series are identical {*X*(*t*_1_) = *X*(*t*_2_) = *X*(*t*_3_) = *X*(*t*_4_) = *X*(*t*_5_) = *A*_*i*_|*i* ∈ (1, …, 5)}, the group of neurons that satisfy *s*_*i*_(*t*_*k*_) ≥ η is the “winner,” and the weights associated with the winning neuron are enhanced via training to yield the same output vector as in the HEBLR. Each distribution of the synapse weight vector has a one-peak distribution to approach the input vector, and the distribution sharpens as the training progresses. In contrast, the distribution in the same contextual input sequences in the STLR has a peak in the distribution of positive regions, which is similar to the HEBLR. Both distributions are mainly created by the “enhancement” subset, which train the same synaptic weight many times in the direction of the input vectors.

For an input vector sequence whose differences between the input vectors have *HD* = 10, the common elements among the input vectors are strengthened, whereas the different elements among the input vectors are subdued as training progresses in the HEBLR. Thereafter, the distribution of the synaptic weight vector converges to a one-peak distribution depending on the common elements between the input vectors. In contrast, in the STLR, there are multiple peaks in the distribution of positive regions. These multiple peaks can be interpreted as the effect of learning by the different input vectors. When different contextual input sequences are trained, the “enhancement” subset creates new distributions around each of the input vectors. Therefore, new synaptic weights corresponding to each input are enhanced and form multiple peaks. This mechanism is the crucial difference between the HEBLR and STLR, and as well as the reason for why the STLR can discriminate similar input sequences and also have excellent pattern discrimination capability.

### 5.2. Verification Through Simulation

We applied input sequences with spatially similar patterns as in natural events to a feed-forward neural network and characterized the learning rules of the HEBLR and STLR by simulation. We set up the input vectors to have a difference of 10 bits between each other (HD = 10), where the cosine distance between each input pattern is approximately 0.93. The synaptic weight vectors are trained to approach this new input axis when the input vector arrives. New synaptic weight vector groups near the input vectors are added by the learning rules, and the synaptic weight vector groups that are far from the input vectors deviate.

The HEBLR algorithm reinforces the synaptic weight vectors in a manner that reinforces the common vector elements of the five input vectors. Therefore, the common synaptic weight vectors are organized. The STLR training for different contextual inputs forms a multimodal distribution of synaptic weight vectors (Figure 5B, lower right panel). This result indicates that the STLR has features that are highly sensitive to different elements among the input vectors. The STLR training algorithms develop the synaptic weight vector distribution in the opposite direction of the input vector. The STLR can also preserve unlearned weight vectors to learn the differences between the next incoming inputs.

### 5.3. STLR and Synaptic LTP/LTD With the Change in Ca^2+^

STLR is a learning rule based on the results of physiological (in vitro) experiments in which the coincidence of neural activity between input cells was controlled (Tsukada et al., 1996, 2007). Furthermore, STLR learns independently of posterior cell firing. This learning rule changes the induction of LTP/LTD depending on the degree of coincidence of neural activity between inputs. Therefore, STLR is closely related to LTP/LTD in physiological experiments.

It is well-known that NMDA receptors, a type of glutamate, play an important role in the induction of LTP in the CA1 area of the hippocampus and in the visual cortex; NMDA receptors are blocked by Mg^2+^ when the postsynaptic membrane potential is at rest, but are depolarized by high-frequency or associative inputs for prolonged periods of time. The NMDA receptor is blocked by Mg^2+^ when the postsynaptic membrane potential is quiescent, but when depolarization is prolonged by a high-frequency or associative input, the Mg^2+^ block is removed and large amounts of Ca^2+^ flow into the post-synapse from NMDA receptor-intrinsic channels. This increase in Ca^2+^ triggers a cascade of biochemical changes that cause long-term changes in transmission efficiency.

It has been clarified that Ca^2+^ is also involved in the induction of LTD. According to the experiment in which a Ca^2+^ chelator was injected into layer II/III neurons to chelate Ca^2+^ at the postsynaptic site in a rat visual cortex section specimen, Ca^2+^ chelated neurons that should cause LTP by the intensity and frequency of titanus input induced LTD (Kimura et al., 1990). This explanation of switching between LTP/LTD demonstrates the validity of the Ca^2+^ binding hypothesis of Ca^2+^/calmodulin-dependent protein kinase II (CaMKII) (Lisman, 1989) and protein dephosphorylating enzyme (Goto et al., 1985; Funauchi et al., 1992).

The retrograde messengers that cause transmitter release from the posterior to anterior synapses can induce LTP/LTD depending on the activity of the presynaptic terminal (Bliss and Collingridge, 1993). The synaptic-induced increase in Ca^2+^ activates various protein kinases and causes the synthesis of retrograde messengers. After entering the synaptic cleft, these synthesized retrograde messengers act on the presynaptic terminal to induce LTP when the terminal is activated, or LTD when it is not. These physiological experiments suggest that the change in Ca^2+^ in the postsynapse triggers LTP/LTD.

It is assumed that in STLR, LTP/LTD is switched by changes in Ca^2+^. The size of the coincidence coefficient (*I*_*ij*_) between inputs changes the triggering of LTP/LTD (Equation 11). This learning rule is consistent with the results of physiological experiments (in vitro) in which phase congruency between input cells was controlled (Tsukada et al., 1996, 2007), and is also consistent with the results that LTP/LTD changes with the “common source” (Silkis, 1996). In addition, although omitted for simplicity, the STLR shows that LTP/LTD changes in response to past input history (Tsukada and Pan, 2005). In other words, the STLR produces LTP/LTD depending on the Ca^2+^ change caused by “common source” and “time history.” This is consistent with physiological experiments wherein the LTP and LTD are not dependent on the absolute size of Ca^2+^ but on the relative one (Abraham and Bear, 1996; Grassi et al., 1996; Silkis, 1996). From this point of view, the BCM model of floating thresholds (relative) by Bienenstock and colleagues, the unitary Hebbian modification rules by Silkis (1998), the learning rules by Frégnac (1991) and Tamura et al. (1992), and the hypothesis of Mayford et al. (1995) are also consistent with our learning rule.

In this study, we compared the characteristics of the HEBLR and STLR through algorithmic examination and simulation. The results suggested that the HEBLR and STLR have contradictory features of self-organization for series of inputs: pattern completion for the HEBLR and pattern discrimination for the STLR. The fact that both learning rules coexist in the hippocampus allows us to understand the remarkable mechanism of self-organization of biological memory neural networks. There is an interesting result that a single-layer neural circuit with feedforward-feedback excitatory connections can instantly learn the dynamic contextual information by integrating these two learning rules (HEBLR and STLR), and learn the information via one-shot learning with self-similar information compression in the memory system (Tsukada et al., 2016, 2018). This result might be one reason why our brains have adopted these two contradictory learning rules during the course of evolution. Our series of studies will contribute to the design of artificial intelligence and can be a promising method for information processing principles such as automatic driving systems that must learn in an external environment in a short period.

## Data Availability Statement

The original contributions presented in the study are included in the article, further inquiries can be directed to the corresponding author.

## Author Contributions

The conception of the study was conducted by HT and MT. HT and MT participated in the design and modeling of the study, drafted and revised the manuscript critically for important intellectual content, and read and approved the manuscript. HT simulated and analyzed the data. MT interpreted and supervised the data. All authors contributed to the article and approved the submitted version.

## Conflict of Interest

The authors declare that the research was conducted in the absence of any commercial or financial relationships that could be construed as a potential conflict of interest.
